# Stigmatization of Chinese and Asian-looking people during the COVID-19 pandemic in Germany

**DOI:** 10.1186/s12889-021-11270-1

**Published:** 2021-07-02

**Authors:** Julia E. Koller, Karoline Villinger, Nadine C. Lages, Isabel Brünecke, Joke M. Debbeler, Kai D. Engel, Sofia Grieble, Peer C. Homann, Robin Kaufmann, Kim M. Koppe, Hannah Oppenheimer, Vanessa C. Radtke, Sarah Rogula, Johanna Stähler, Britta Renner, Harald T. Schupp

**Affiliations:** University of Konstanz, 78457 Konstanz, Germany

**Keywords:** Stigmatization, Stigma, Coronavirus, COVID-19, SARS-CoV-2, Pandemic, Disease, Threat, Avoidance, Pathogen

## Abstract

**Background:**

The outbreak and global spread of COVID-19 was accompanied by an increase in reports of stigmatization of Chinese and Asian-looking people. The behavioral immune system provides a framework for stigmatization in response to infectious disease threats. Specifically, stigmatization might increase with rising levels of infectious disease threat. The present study aimed to examine this hypothesis during the early phase of the COVID-19 pandemic.

**Methods:**

As part of the “EUCLID” project (https://euclid.dbvis.de), a total of 5011 persons from Germany were surveyed via an online-questionnaire between February 2^nd^ and April 3^rd^, 2020, covering the progression of the COVID-19 pandemic over three time periods which were defined by critical events.

**Results:**

There was no evidence for an increase in the stigmatization of Chinese and Asian-looking people across three topics, that is personal proximity, air travel, and medical measures upon arrival from China.

**Conclusions:**

The present findings provide good news in that participants showed an adaptive response to the infectious disease threat rather than displaying increased stigmatization. Further research is necessary to specify the conditions that increase the risk of stigmatization in response to infectious disease threats.

**Supplementary Information:**

The online version contains supplementary material available at 10.1186/s12889-021-11270-1.

## Background

The coronavirus SARS-CoV-2, which was first reported in December 2019 in Wuhan, China, spread rapidly around the world, causing the deaths of millions of people worldwide [1]. This global spread was accompanied by a reported surge in the stigmatization of Chinese and Asian-looking individuals [2–6]. Accordingly, individuals who are perceived to look Chinese or more generally Asian may be stigmatized based on their appearance, leading to avoidance behaviors and social exclusion [7]. In response to the reports of stigmatization, the Centers for Disease Control and Prevention felt compelled to try to reduce the stigma by emphasizing that “No single person or group of people are more likely than others to spread COVID-19” [8].

Stigmatization is often conceptualized from an evolutionary perspective (e.g., [9–12]). In the evolutionary disease-avoidance model, stigmatization is conceived as the result of a cognitive adaptation for disease-avoidance which developed in response to problems within ancestral environments [10–12]. Perceived vulnerability to disease represents one of several conceptually distinct pathways which link specific adaptation problems of disease-avoidance to stigmatization [9, 10]. Specifically, stigmatization has been linked to infectious disease threats, such as pandemics both historically [13] and from a psychological perspective through disease-avoidance mechanisms, which are also often referred to as the human “behavioral immune system” (BIS; [11, 14]). The BIS enables people to identify and avoid cues of infection, thereby presumably minimizing their risk of becoming infected. However, since the risk of infection needs to be inferred, cue detection may be flawed. To avoid the costly consequences of detection errors and the burden of an infection for the biological immune system, there is a tendency to interpret harmless characteristics (e.g., facial anomalies) as infection cues [11, 14, 15]. Furthermore, outgroup membership may lead to avoidance and stigmatization (e.g., [11, 16]) when people perceive that group to be associated with an infectious disease [17]. Stigmatization has been shown to be especially prevalent when people feel vulnerable to infection [11, 14, 15]. The outbreak of the COVID-19 pandemic may have activated the BIS in the general population [18, 19], thereby increasing stigmatization [11, 20, 21]. Reports and initial studies suggest that the outbreak of the COVID-19 pandemic may indeed have led to an increase in stigmatization of Chinese and Asian-looking people, who are perceived by some people as posing an infection risk (e.g., [3–6, 22, 23]). Several studies also observed an increase in the use of stigmatizing language on the internet during the early phase of the pandemic, which supports this notion (e.g., [24–26]).

The present study investigated changes in the stigmatization of Chinese and Asian-looking people over three time periods, which mark the progression of COVID-19 through critical events in Germany. While stigmatization is a multifaceted construct, the desire to avoid a certain group of people is at its core [27]. Accordingly, stigmatization may lead to avoidance and exclusion on interpersonal and societal levels. Thus, on an interpersonal level, we probed stigmatization related to worry about being in personal proximity to Chinese and Asian-looking people. On a societal level, we assessed people’s approval to single out and exclude Chinese and Asian-looking people via governmental measures regarding air travel restrictions and medical measures upon arrival from China. An overall increase in stigmatization was hypothesized over time with increased infectious disease threat.

## Methods

The present study focused on a selection of data collected in Germany between February 2^nd^ and April 3^rd^, 2020, via google forms and Qualtrics. It is part of the “EUCLID” project, which tracks changes in subjective health, perceptions, expectations, and behavior related to the COVID-19 pandemic over time and in different countries (https://euclid.dbvis.de). The questions used in this study were specifically developed for the “EUCLID” project (Additional file 1). Data were stored in anonymized form and treated confidentially. In March 2020, the ethics committee of the University of Konstanz (ID # 07/2020) approved that the study adhered to the guidelines of the German Psychological Society and the declaration of Helsinki. Participants gave written informed consent to participate prior to their participation.

Participants living in Germany and older than 18 years of age were recruited via social media (Facebook, Twitter), email lists using a snowball system, and Prolific Academic. Participants could either take part in a lottery for a 25€ Amazon voucher or received a payment from Prolific Academic. A total of 5443 participants were recruited in Germany, of whom 432 were excluded due missing core variables or failed attention checks to ensure a high data quality. A final sample of 5011 participants (75.35% women) with a mean age of 33.37 years (*SD* = 13.19, 18–90 years) was included in the analyses. Overall, 44.32% indicated being employed or self-employed, while 45.90% were in training or education. While participants came from all German federal states, the majority were from Baden-Wuerttemberg (35.78%), North Rhine-Westphalia (17.30%), and Bavaria (12.09%).

**Fig. 1:**
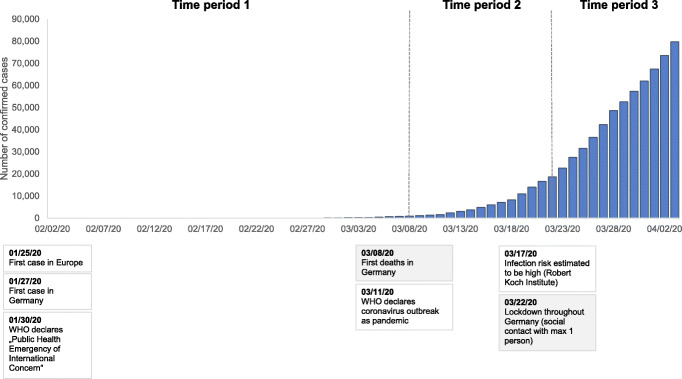
Time periods as defined by critical events related to COVID-19 in Germany, global COVID-19-related events, and cumulative SARS-CoV-2 cases in Germany per day. Data source: [28]

Three time periods were defined based on critical COVID-19-related events in Germany and reflecting increasing threat levels (see Fig. 1): early phase (T1: 02/02–03/07/20, *n* = 1143), after the first deaths (T2: 03/08–03/21/20, *n* = 1462) and during the lockdown (T3: 03/22–04/03/20, *n* = 2406).

Personal proximity reflects an important aspect of disease-avoidance, as direct physical contact with potentially infected individuals poses a risk of disease transmission [9, 29–31]. As with potentially infected individuals, direct physical contact with stigmatized people may elicit discomfort and thereby facilitate avoidance on a personal level. Thus, at the interpersonal level, stigmatization was assessed by asking the participants to indicate their “worry about shaking hands with a Chinese-looking person” and a control question “worry about shaking hands with a recent traveler from China” (i.e., someone who has arrived in Germany from China in the last 4 weeks). A third question asked participants to estimate their “worry about a COVID-19 quarantine station in one’s own city”. This item was selected based on the finding that being in close proximity to potentially infected individuals may elicit fear, even without direct physical contact [31]. Furthermore, people who have been quarantined because they are potentially infected pose a high risk of transmitting the disease [32]. Thus, the quarantine control question assessed worry at a general level, thereby facilitating the interpretation of possible stigmatization effects against Chinese and Asian-looking people. Worry was measured on a 5-point scale ranging from 1 (not at all worried) to 5 (very worried).

To probe for societal levels of stigmatization, two further topics focused on the agreement with governmental measures, namely restricting air travel and imposing medical measures upon arrival from China. At the time of the study, such measures were widely discussed in the German public and some measures such as air travel restrictions may in fact be effective to contain the spread of the virus [33]. However, agreement with measures specifically for Chinese but not for other people, especially if they had recently been to China at a time when it had the highest number of coronavirus cases, might indicate stigmatization. Accordingly, we asked participants to indicate how much they would agree with an “air travel ban for Chinese people”, which was compared to their agreement with a “suspension of air travel to and from China”. Furthermore, agreement with a “suspension of international air travel” was assessed to obtain the level of agreement with a suspension of air travel in general. Agreement was assessed on a 4-point scale ranging from 1 (strongly disagree) to 4 (strongly agree).

Similarly, with regard to medical measures, stigmatization was explored by comparing agreement with “compulsory medical examinations for Chinese passengers arriving from China” and agreement with “compulsory medical examinations for European passengers arriving from China”. A further question asked whether there should be “a general quarantine requirement for people arriving from China”. Agreement was again assessed on a 4-point scale.

A 3 (item) × 3 (threat level) mixed MANOVA was calculated for each of the three topics. ANOVAs and t-tests with Bonferroni correction were calculated to follow-up on significant effects. As effect size measures, eta squared (*η*^2^) and partial eta squared (*η*_*p*_^2^) were computed with cut-off values of .01, .06, and .14 for small, medium, and large effects as proposed by Cohen [34].

## Results

### Personal proximity

**Fig. 2:**
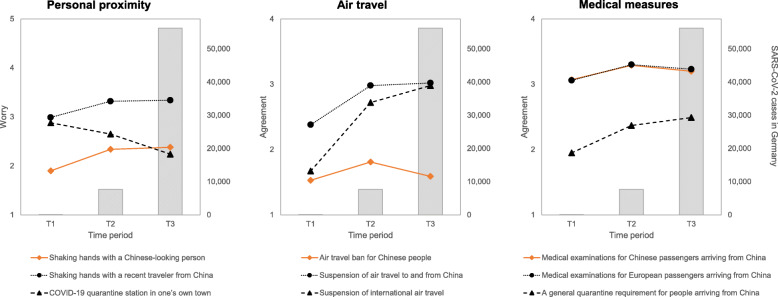
Personal proximity (left panel; scale 1–5), air travel (middle panel; scale 1–4), and medical measures upon arrival from China (right panel; scale 1–4) over the three time periods are shown on the left y-axis. Average SARS-CoV-2 cases per time period in Germany are displayed on the right y-axis (grey). Items exploring stigmatization relative to the control items are highlighted in orange. Data source for SARS-CoV-2 cases: [28]

As shown in Fig. 2, the comparison between shaking hands with a Chinese-looking person and a recent traveler from China provided little evidence for stigmatization. There was a significant main effect for personal proximity, (*F* (2, 9960) = 1453.19, *p* < .001, *η*_*p*_^2^ = .23). Post-hoc tests indicated that participants felt most worried about shaking hands with a recent traveler from China (*M* = 3.25, *SD* = 1.22), followed by having a COVID-19 quarantine station in one’s own city (*M* = 2.50, *SD* = 1.24), and finally shaking hands with a Chinese-looking person (*M* = 2.26, *SD* = 1.23; *p*s < .001).

Furthermore, a significant interaction of personal proximity x threat level was observed (*F* (4, 9960) = 181.78, *p* < .001, *η*_*p*_^2^ = .07), which was primarily driven by worry about having a COVID-19 quarantine station in one’s own city (see Fig. 2). Surprisingly, worry about having a COVID-19 quarantine station in one’s own city decreased with increasing threat levels (*F* (2, 4997) = 122.86, *p* < .001, *η*^2^ = .05; post-hoc: *p*s < .001). In contrast, worry about shaking hands with a Chinese-looking person (*F* (2, 4993) = 64.10, *p* < .001, *η*^2^ = .03) and a recent traveler from China (*F* (2, 4994) = 35.81, *p* < .001, *η*^2^ = 0.01) followed similar trajectories with a small increase from T1 to T2 (*p*s < .001), and no change from T2 to T3 (*p*s = 1.00).

### Air travel

As with personal proximity, findings regarding air travel did not provide evidence for stigmatization (see Fig. 2). Specifically, the main effect of air travel (*F* (2, 9740) = 3947.72, *p* < .001, *η*_*p*_^2^ = .45) indicated that participants most commonly agreed with a suspension of air travel to and from China (*M* = 2.86, *SD* = 0.94), followed by a suspension of international air travel (*M* = 2.60, *SD* = 0.99), and lastly an air travel ban for Chinese people (*M* = 1.64, *SD* = 0.89, *p*s < .001).

A significant interaction of air travel x threat level was also observed (*F* (4, 9740) = 374.44, *p* < .001, *η*_*p*_^2^ = .13). Contrary to an expected increase in stigmatization, agreement with an air travel ban for Chinese people (*F* (2, 4877) = 37.75, *p* < .001, *η*^2^ = .02) only increased slightly from T1 to T2 (*t* (4877) = − 7.89, *p* < .001) and decreased from T2 to T3 (*t* (4877) = 7.23, *p* < .001), returning to the initial level (*t* (4877) = − 1.93, *p* = .163). While agreement with a suspension of air travel to and from China (*F* (2, 4887) = 209.08, *p* < .001, *η*^2^ = .08) also increased from T1 to T2 (*t* (4887) = − 16.66, *p* < .001), it remained stable between T2 and T3 (*t* (4887) = − 1.40, *p* = .484). A different pattern was seen for agreement with a suspension of international air travel (*F* (2, 4896) = 948.15, *p* < .001, *η*^2^ = .28), which increased substantially with increasing threat level (*p*s < .001).

### Medical measures upon arrival from China

As shown in Fig. 2, agreement with compulsory medical examinations for Chinese and European passengers was highly similar across the three time periods, again, providing no evidence for stigmatization. There was a main effect for medical measures (*F* (2, 9728) = 4273.68, *p* < .001, *η*_*p*_^2^ = .47). Post-hoc tests indicated substantially lower agreement with a general quarantine requirement for people arriving from China (*M* = 2.33, *SD* = 0.99) than medical examinations for European (*M* = 3.21, *SD* = 0.82, *p* < .001) and Chinese (*M* = 3.20, *SD* = 0.83, *p*s < .001) passengers. Furthermore, agreement was slightly higher for medical examinations for European compared with Chinese passengers (*p* < .001).

A small effect of medical measures x threat level emerged (*F* (4, 9728) = 67.00, *p* < .001, *η*_*p*_^2^ = .03). Agreement with medical examinations for both Chinese (*F* (2, 4877) = 22.87, *p* < .001, *η*^2^ = .01) and European passengers (*F* (2, 4886) = 28.90, *p* < .001, *η*^2^ = .01) arriving from China increased slightly between T1 and T2 (*p*s < .001), followed by a small decrease from T2 to T3 (*p*s < .05). Furthermore, agreement with a general quarantine requirement for people arriving from China increased slightly with increasing threat levels (*F* (2, 4883) = 121.60, *p* < .001, *η*^2^ = .05; post-hoc: *p*s ≤ .001).

## Discussion

The present study examined the hypothesis that the stigmatization of Chinese and Asian-looking people increases with rising levels of infectious disease threat. However, measures of stigmatization on interpersonal and societal levels yielded no support for this hypothesis. Specifically, worry about shaking hands with a Chinese-looking person did not increase with rising threat levels and was notably lower than shaking hands with a recent traveler from China. Furthermore, participants were more inclined to support a suspension of air travel to and from China, and at later time periods even a suspension of international air travel, rather than a travel ban specifically for Chinese people. In addition, there was broad agreement that both Chinese and European passengers should be medically examined upon arrival from China. With regard to worry of interpersonal contact and agreement with governmental measures, the findings of the present study do not support the notion derived from the BIS that stigmatization of foreign outgroups increases with infectious disease threats such as those that occur in times of pandemics.

The present findings appear to contradict several reports which suggest that the outbreak of COVID-19 has led to increased stigmatization of Chinese and Asian-looking people, possibly triggered by feelings of fear (e.g., [2–4, 6]). More direct evidence is provided by Sorokowski and colleagues [35], who showed that higher exposure to coronavirus-related news was associated with higher levels of anxiety and negative attitudes towards Italians, but not towards Chinese people or other nationalities. The specificity of these findings was discussed with respect to the current epidemiological situation and associated media coverage, as Italy was severely affected by COVID-19 at the time of the study [35]. In support of this notion, Xu and colleagues [5] showed how stigmatization of different geographic target groups spread within China, neighboring countries (i.e., Hong Kong and Taiwan), and other global regions as a function of COVID-19 prevalence. Furthermore, the activation of the BIS in response to an infectious disease threat may only lead to negative attitudes toward an outgroup when people perceive that this outgroup is associated with an infectious disease [17, 36]. During data collection for the present study, the situation in China improved around the end of February 2020 [37], providing a possible explanation of why the increase in SARS-CoV-2 cases was not associated with increased stigmatization. However, even an exploratory examination of the data preceding the improvements in China found little evidence for changes in stigmatization. It also seems possible that an increase in stigmatization with increasing threat level was not found because of the contrasting dynamics of the COVID-19 pandemic in China and Germany. Specifically, in times of high threat in China, the number reported infections in Germany was low. However, similar to our findings, a recent study with US and Singaporean samples observed that while priming the salience of the pandemic increased the support for travel bans, specifically for high-risk countries (i.e., China and Italy), no increase in xenophobia was observed [38]. Furthermore, a study relating objective threat levels (i.e., the number of confirmed COVID-19 cases in different regions of the UK) to outgroup distancing and prejudice also provided rather mixed results [39].

There are several other possible explanations as to why no increase in stigmatization was observed in the present study. It has been suggested that engagement in disease avoidance may involve different modules which build upon perceptual and cognitive processes [40]. The present study relied on abstract labeling (i.e., “Chinese-looking person”), and it is possible that different results would have been observed by presenting concrete stimuli such as photographs. In addition, the present study relied on self-reports, and the possibility of social desirability needs to be considered when examining sensitive issues. However, even the topic on medical measures indicated low levels of stigmatization, although the issue was assessed indirectly by contrasting response patterns. Furthermore, since the cues activating the BIS may be learned in the socio-cultural context (i.e., through media and political discourse), ethnic stereotypes associated with disease may vary across countries [41]. In addition, due to the sampling method, the study sample is not representative of the German population (e.g., the mean age is younger at 33.37 compared to 44.25 within the general population; [42]), and illiterate individuals and people without access to the Internet were not able to participate in the study. Thus, the present findings await replication based on representative samples and future research may consider potential effects of age on stigmatization. Notwithstanding these caveats, the present results provide reason for cautious optimism in that participants mostly showed adaptive responses (e.g., favoring a suspension of air travel to and from China) rather than exhibiting more stigmatization (e.g., regarding an air travel ban for Chinese people) in times of increased infectious disease threat.

## Conclusions

Previous research on infectious disease threat and stigmatization primarily relied on experimental manipulations of threat levels (e.g., [11, 16, 19, 38, 43]). Expanding research on real threat situations (e.g., [39, 44, 45]), the present study investigated stigmatization in relation to the newly emerging COVID-19 pandemic as a function of increasing threat levels (i.e., a rising number of infections). Surprisingly, the findings do not yield support for an increase in the stigmatization of Chinese and Asian-looking people as the threat level increased over the early phases of the pandemic in Germany. Further research is needed to determine the conditions leading to increases in stigmatization in response to infectious disease threats.

It is important to note that this study explicitly does not suggest that there has been no stigmatization in relation to the pandemic. While the majority of participants appeared to display adaptive responses, there was a small proportion of participants with responses which did indicate stigmatization. Accordingly, the findings from this study should not be used as an excuse to trivialize stigmatization and its negative impact on the people who are affected by it.

## Supplementary Information

**Additional file 1.** Selection of questions from the “EUCLID” project. A selection of questions which were developed for the “EUCLID” project and which are used in the present study.

## Data Availability

The datasets used and/or analyzed during the current study are available from the corresponding author on reasonable request.

## Abbreviations

BIS: Behavioral immune system
COVID-19: Coronavirus disease 2019
SARS-CoV-2: Severe acute respiratory syndrome coronavirus 2
