# Supplementary material for: Stigmatization of Chinese and Asian-looking people during the COVID-19 pandemic in Germany
Source: BMC Public Health. 2021 Jul 2;21:1296. doi: 10.1186/s12889-021-11270-1 (PMC8253234; doi:10.1186/s12889-021-11270-1)
Supplement: Supplementary file 1 — Additional file 1. Selection of questions from the “EUCLID” project. A selection of questions which were developed for the “EUCLID” project and which are used in the present study. [file 12889_2021_11270_MOESM1_ESM.pdf]

Questions

Coronavirus survey

Electronic survey on the current coronavirus situation

Universität Konstanz

EUCLID

Electronic survey on the current coronavirus situation

copyright 2020 University of Konstanz all rights reserved

Regarding an infection with the coronavirus, how WORRIED do you feel ...

	not at all worried	slightly worried	moderately worried	worried	very worried
...shaking hands with a person who looks Chinese?	○	○	○	○	○
...shaking hands with a person who arrived from China during the last 4 weeks?	○	○	○	○	○
...a quarantine station for coronavirus patients being set up in your city?	○	○	○	○	○

With regard to air travel: How much do you agree with the following statements?

	strongly disagree	rather disagree	rather agree	strongly agree
International air traffic should be suspended.	○	○	○	○
Air traffic from and to China should be suspended.	○	○	○	○
Air travel should generally be prohibited for Chinese people.	○	○	○	○
European passengers arriving from China should be medically examined upon arrival at the airport.	○	○	○	○
Chinese passengers arriving from China should be medically examined upon arrival at the airport.	○	○	○	○
People arriving from China should be quarantined regardless of wether or not a coronavirus infection is suspected.	○	○	○	○

copyright 2020 University of Konstanz all rights reserved

Powered by Qualtrics
